# Antiviral Activity of Silver, Copper Oxide and Zinc Oxide Nanoparticle Coatings against SARS-CoV-2

**DOI:** 10.3390/nano11051312

**Published:** 2021-05-17

**Authors:** Padryk Merkl, Siwen Long, Gerald M. McInerney, Georgios A. Sotiriou

**Affiliations:** Department of Microbiology, Tumor and Cell Biology, Karolinska Institutet, SE-17177 Stockholm, Sweden; padryk.merkl@ki.se (P.M.); siwen.long@ki.se (S.L.)

**Keywords:** nanosilver, antimicrobial, surface modification, filtration

## Abstract

SARS-CoV-2 is responsible for several million deaths to date globally, and both fomite transmission from surfaces as well as airborne transmission from aerosols may be largely responsible for the spread of the virus. Here, nanoparticle coatings of three antimicrobial materials (Ag, CuO and ZnO) are deposited on both solid flat surfaces as well as porous filter media, and their activity against SARS-CoV-2 viability is compared with a viral plaque assay. These nanocoatings are manufactured by aerosol nanoparticle self-assembly during their flame synthesis. Nanosilver particles as a coating exhibit the strongest antiviral activity of the three studied nanomaterials, while copper oxide exhibits moderate activity, and zinc oxide does not appear to significantly reduce the virus infectivity. Thus, nanosilver and copper oxide show potential as antiviral coatings on solid surfaces and on filter media to minimize transmission and super-spreading events while also providing critical information for the current and any future pandemic mitigation efforts.

## 1. Introduction

Severe acute respiratory syndrome coronavirus 2 (SARS-CoV-2) is an enveloped RNA virus that causes the respiratory illness coronavirus disease 2019 (COVID-19) and has led to over 3 million deaths to date. The World Health Organization (WHO, Geneva, Switzerland) suggests that droplet, contact, fomite (contaminated surfaces) and airborne (aerosol) transmissions are considered to be potential routes of infection and continues to recommend both droplet and contact precautions for those people caring for COVID-19 patients [1]. When persons infected with the SARS-CoV-2 novel coronavirus cough or sneeze, they may emit tiny droplets that contain the virus, resulting in the contamination of their surrounding surfaces and risking transmission to other persons via fomites. SARS-CoV-2 remains stable on surfaces for many hours after its arrival there, providing a potential pathway for infecting healthy persons. A study [2] of March 2020 showed that there are large variations in the stability of this virus on different surfaces (plastic, stainless steel, cardboard, copper), and this has prompted researchers to explore whether modifying the physicochemical properties of surfaces (including face masks) reduces the stability of SARS-CoV-2 and, thus, its transmission to healthy persons [3]. Furthermore, in the case of aerosol transmission, the air filtration and disinfection efficiency of air filtration/circulation units plays an important role in minimizing the virus transmission, especially in areas with no external air supply (e.g., public transportation vehicles) or in clinics and nosocomial settings [4].

In recent years, a growing interest has been seen in the use of self-decontaminating surface materials as additional safety measures towards preventing disease transmission, for example using surface coatings with Ag [5], CuO [6] and ZnO [7] nanoparticles. Nanosilver (Ag) is perhaps the most studied antibacterial nanomaterial and its antimicrobial mechanism is largely attributed to the release of Ag^+^ ions upon contact with aqueous systems [8,9]. Nanosilver kills bacteria by targeting the bacterial cell envelope and by generating reactive oxygen species [10]. Due to its high antibacterial activity, nanosilver has been explored as an antibiofilm coating, while its antibacterial activity has also been exhibited against fungi and viruses including SARS-CoV-2 [11,12]. Similarly, both CuO and ZnO nanoparticles have been studied as antibacterial materials, but to a lesser extent than nanosilver. The aim of this study is to modify surfaces with such antiviral nanoparticle coatings and compare their ability to neutralize the novel coronavirus upon landing on these surfaces. These nanostructured coatings are developed using a scalable and reproducible nanomanufacturing process, namely, flame aerosol nanoparticle deposition, to assist facile integration in the manufacturing of air filter components and rapid translation in industry. Coatings of Ag, CuO and ZnO on both flat solid substrates (glass) as well as porous filter media (glass fiber filters and FFP3 filters) are produced with the aim of studying their antiviral activity against SARS-CoV-2 under identical incubation conditions.

## 2. Materials and Methods

### 2.1. Nanoparticle Film Assembly and Characterization

Nanoparticles were produced by flame spray pyrolysis (FSP), an aerosol synthesis technique described elsewhere [13]. In brief, the liquid precursor solutions at a concentration of 0.3 M for the production of CuO and ZnO nanoparticles were prepared by dissolving appropriate quantities of copper (II) nitrate (99–104%, Sigma Aldrich, Stockholm, Sweden) in a 1:1 ethanol (absolute, VWR, Spånga, Sweden):2-ethylhexanoic acid (≥99%, Sigma Aldrich) mixture and zinc acetate (97%, Alfa Aesar, Kandel, Germany) in a 1:1 methanol (ACS reag., VWR):2-ethylhexanoic acid mixture, respectively. For Ag/SiO_2_ nanoparticle production, silver acetate (99%, Alfa Aesar) was dissolved in a 1:1 mixture of acetonitrile (99.8%, Sigma Aldrich):2-ethylhexanoic acid under reflux at 70 °C to achieve a 0.3 M solution with subsequent addition of hexamethyldisiloxane (≥98%, Sigma Aldrich) to achieve a 50 wt % content of SiO_2_ in the nanoparticle product. The precursor solutions were fed through a capillary from a 50 mL syringe (SGE Analytical Science, Milton Keynes, United Kingdom) by a syringe pump (New Era Pump Systems, Inc., Farmingdale, United States of America) at a rate of 5 mL/min and dispersed by 5 L/min (EL-FLOW Select, Bronkhorst, Ruurlo, Netherlands) of oxygen (5.0, Strandmöllen AB, Ljungby, Sweden) with a pressure drop at the nozzle of 1.8 bar. The spray was ignited by a pre-mixed methane/oxygen (5.5, Linde Gas AB, Solna, Sweden) flamelet with flow rates of 1.5 L/min and 3.2 L/min, respectively. Nanoparticles were collected on a glass fiber filter (Hahnemühle, Dassel, Germany) further downstream with the aid of a vacuum pump (Mink MM 1144, Busch Vakuumteknik AB, Mölynlycke, Sweden). Deposition of nanoparticle metal and metal oxide films onto glass substrates was performed by positioning the flame directly below a water-cooled substrate holder as described by Tricoli et al. [14]. The height of the substrate above the nozzle during deposition was 20 cm, and the deposition time was 15 s. Following deposition of the metal and metal oxides on the glass substrates, an in situ annealing step was performed in which a pure ethanol flame was pumped for 15 s at a rate of 12 mL/min and dispersed by 3 L/min of oxygen, while the water cooling of the substrate holder was turned off. The XRD diffractograms of the metal and metal oxide nanocoatings were collected using a Rigaku MiniFlex 600, CuKα radiation and analyzed with the PDXL2 software (Rigaku, Neu-Isenburg, Germany). The SEM micrographs were collected using a Phenom Pharos (Thermo Fisher, Waltham, United States of America). The TEM images were collected using a Talos 120C G2 (Thermo Fisher) with a 120 kV LaB_6_ beam voltage.

### 2.2. Cells and Virus

Vero-E6 cells (ATCC-CRL-1586) were maintained in Dulbecco’s Modified Eagle Medium (Sigma, Munich, Germany) supplemented with 10% fetal calf serum and 1% penicillin–streptomycin and cultured at 37 °C in a humidified incubator with 5% CO_2_. SARS-CoV-2, isolated from the first Swedish patient, was received from the Public Health Agency of Sweden.

### 2.3. Setup for Virucidal Activity of Nanoparticles

Coated with the nanocoatings (Ag/SiO_2_, CuO and ZnO) or uncoated (control group) 22 × 22 mm glass slides were placed into a 6 cm dish, and a 50 μL SARS-CoV-2 droplet was applied in the center of the slide. The droplet was immediately covered with an uncoated glass slide to spread the virus over the whole area. After incubation for different lengths of time (5, 30, 120 min) at room temperature, 1950 μL of 1 X phosphate buffered saline (PBS) was added to the coverslip. The cover slide was lifted and flipped over, and the two virus-exposed sides were washed gently by pipetting 10 times. Then, the whole washings were collected and stored at −80 °C for titer determination.

### 2.4. Plaque Assay

Confluent Vero-E6 cells in 24-well plates were washed with PBS, and twofold serial virus dilutions of SARS-CoV-2 were added in 200 μL minimal essential medium (Sigma, Munich, Germany) supplemented with 0.2% bovine serum albumin, 2 mM L-glutamine and 20 mM HEPES with periodic shaking for 1 h 37 °C. Virus solutions were then removed, and cells were washed with PBS before addition of 1 mL of prewarmed overlay (2% methylcellulose: propagation media containing 2% FBS = 2:3). At 48–72 h post infection, cells were fixed with 4% formaldehyde and stained with crystal violet solution after removal of the overlay, and plaques were manually quantified. The determination of all virus titers was performed in triplicate.

## 3. Results and Discussion

**Figure 1:**
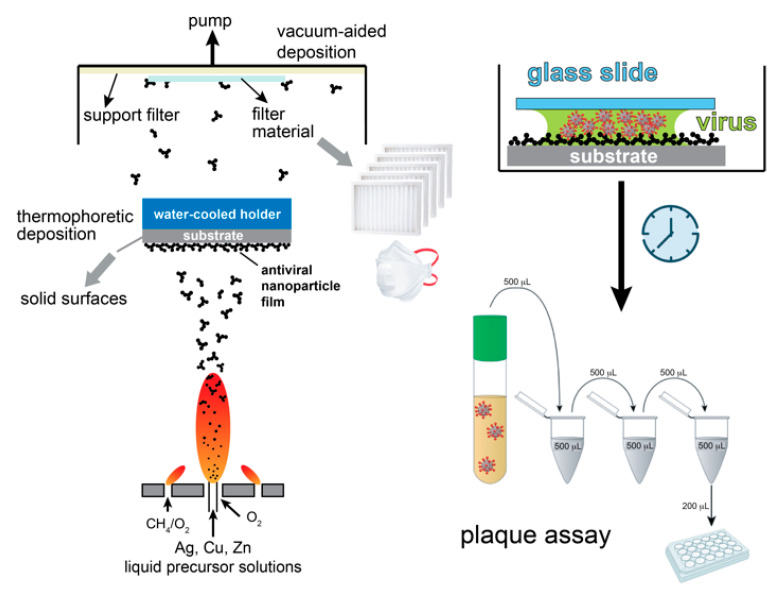
Schematic of the flame aerosol deposition process of antiviral nanoparticle coatings on solid flat substrates as well as on porous filter materials. The as-prepared nanoparticle coatings are then incubated with SARS-CoV-2, and their antiviral activity is examined by the plaque assay.

The process for the production and in situ deposition of anti-viral nanoparticles on selected substrates is shown schematically in Figure 1. We utilized an aerosol reactor in which the product nanoparticles were made by flame spray pyrolysis of appropriate metal-containing liquid precursor solutions [15]. In this process, the as-prepared hot aerosol was deposited either by thermophoresis further downstream on the selected solid substrates attached to a water-cooled holder, or by filtration on the selected porous filter materials with the aid of a vacuum pump. The nanoparticle film thickness and porosity were controlled by the deposition duration as well as an optional in situ flame annealing step, in which the film was mechanically stabilized by an impinging particle-free flame [14].

Flame aerosol deposition is a rapid nanomanufacturing process that combines particle production and functional film assembly in a single step and offers great flexibility in material composition [16] as well as in the characteristics of the nanoparticles [17]. The anti-SARS-CoV-2 activity of the nanoparticle films was then examined upon incubation for up to 2 h with the virus at 37 °C and quantified by plaque assay [18].

**Figure 2:**
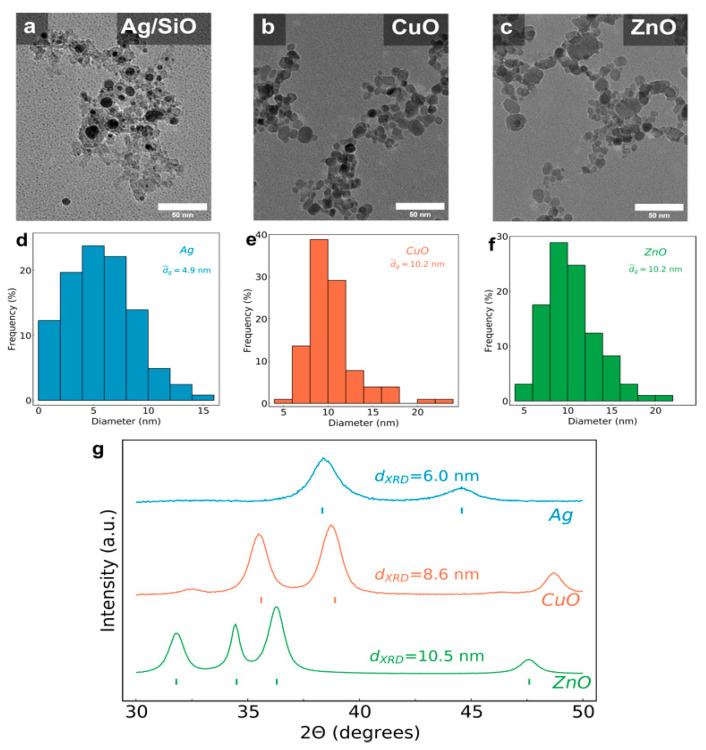
TEM images of the scraped-off nanoparticles from the annealed nanocoatings on the glass substrates for the (**a**) Ag/SiO_2_, (**b**) CuO and (**c**) ZnO samples. (**d**–**f**) Particle size distributions obtained from the TEM images for all three samples. (**g**) X-ray diffraction patterns of the nanocoatings on the glass substrates along with their average crystal size.

Nanosilver particles were made with a support material (nanostructured SiO_2_) to inhibit their full coalescence during their flame synthesis due to the high temperatures in the reactor and the low melting point of Ag [8]. Nanoparticle films on both solid glass flat substrates and porous filter material were fabricated with a deposition duration of 15 s, further highlighting the rapid nature of this process. Figure 2 shows transmission electron microscopy (TEM) images of (a) Ag/SiO_2_, (b) CuO and (c) ZnO nanoparticles that have been recovered from the porous filter media. All three samples exhibit a fractal-like agglomerate structure characteristic of flame-made materials. The primary particle size distributions from these TEM images for all three samples, along with their average particle diameter, are shown in Figure 2d–f for Ag, CuO and ZnO, respectively. The average primary nanosilver particle diameter (d_TEM_) is ~5 nm, while the d_TEM_ sizes from CuO and ZnO are ~10 nm. Figure 2g shows the X-ray diffraction patterns of the as-prepared nanoparticles as collected on a glass fiber filter, along with their average crystal sizes d_XRD_, which are in good agreement with the d_TEM_ values obtained by electron microscopy. The corresponding peak positions for Ag, CuO and ZnO crystal phase compositions are also shown, confirming the purity of the samples.

**Figure 3:**
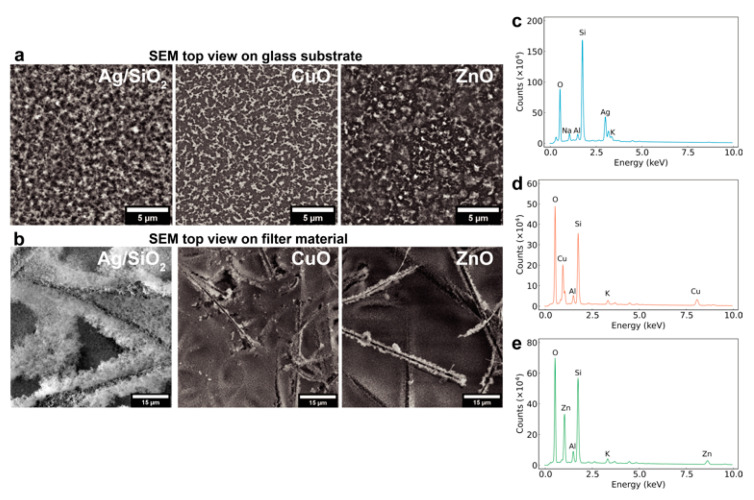
Scanning electron microscope images of all three Ag, CuO and ZnO nanocoatings on (**a**) flat glass substrates and (**b**) porous filter material along with their energy dispersive X-ray spectra in (**c**–**e**), respectively.

Figure 3 shows top-view scanning electron microscope images of the particle films on (a) solid glass substrates and (b) porous filter media. The particle film layers are rather homogeneous on the flat glass substrates and exhibit a nanoporous structure in agreement with nanoparticle films made by aerosol processes [14]. Similarly, the nanoparticles appear to homogeneously coat the fibers of the filter materials as shown in Figure 3b. Figure 3c–e also show electron dispersive X-ray spectra of the particle films as deposited on the porous glass fiber filters that reveal their elemental composition, further confirming the presence of Ag, CuO and ZnO nanoparticles there (Si, Na, Al, K signals originate from the glass fiber filter background).

**Figure 4:**
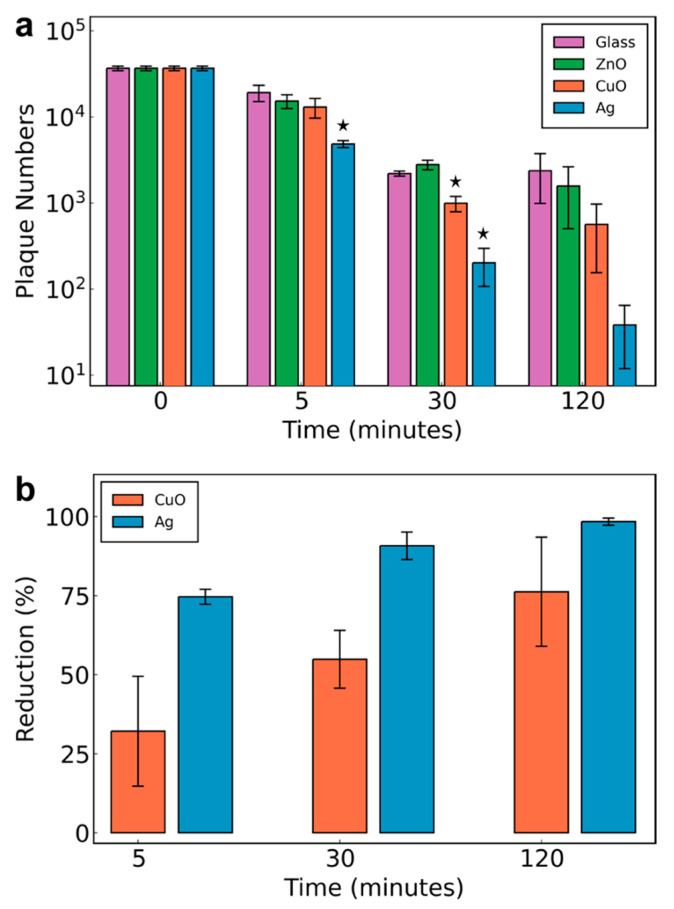
(**a**) SARS-CoV-2 residual viral load as determined by plaque assay on the developed nanocoatings up to 120 min (*n* = 3), * *p* < 0.05, all *p*-values in Supplementary Materials, Table S1. (**b**) Reduction % of SARS-CoV-2 on CuO and Ag/SiO_2_ nanoparticle-coated surfaces as calculated from (**a**); error bars are the standard error of the mean.

The time course of the antiviral capacity of the developed coatings was further examined by plaque assay. A virus-containing droplet was administered to the nanoparticle films, and another plain glass substrate of the same size was placed on top to flatten the drop and avoid evaporation. The antiviral activity model here was designed specifically in order to compare the antiviral capacities of each nanomaterial. After a room temperature incubation for 5, 30 and 120 min, respectively, the top slide was lifted and virus-exposed sides of the two glass substrates were washed with PBS. The washings underwent eight consecutive 2-fold dilutions with the infection media for titer determination (Supplementary Materials Figure S1). Figure 4 shows (a) the virus load (plaque numbers) after 5, 30 and 120 min for all three different nanoparticle films on the glass substrates along with the control sample (pure glass substrate) and (b) the virus reduction % of the CuO and Ag/SiO_2_ nanoparticle-coated surfaces as compared to the virus stability on glass. On pure glass, the viral load appears to decrease rather quickly for the first 30 min, but then remains stable for up to 2 h. The ZnO nanoparticle film does not appear to significantly affect the virus stability over time. However, the CuO nanoparticle film appears to significantly reduce the viral load already after 30 min (54% reduction), and after 120 min it reaches up to 76% reduction. This viral load reduction on CuO surfaces is in agreement with the observations of the approximately 1 h of half-life of SARS-CoV-2 on copper surfaces [2]. Micron-sized particle-based cupric oxide coatings on steel or glass substrate materials also have the capacity to drastically reduce the SARS-CoV-2 infectivity [19], even though the high temperature processing during their fabrication might impose limitations on their employment as coatings on porous filter media. In contrast, the aerosol deposition coating process employed here allows for coating fabrication on a variety of surfaces, including porous filters, as shown in Figure 3b. Most importantly, the Ag/SiO_2_ nanoparticle film exhibits the most drastic effect on the residual viral load already after 5 min with a significant 75% reduction and up to 98% reduction after 120 min, highlighting the potential of nanosilver as an effective antiviral coating. After 120 min, there are no statistically significant values obtained, and this is attributed to the variation of measurements at these conditions. Nonetheless, in the case of nanosilver, the reduction is of two orders of magnitude and not-overlapping error bars indicate that the reduction observed here is in fact large and not an experimental artefact. This is additionally corroborated by the fact that the differences in plaque numbers at both earlier time points were indeed significantly lower, despite the actual reductions being less extensive.

## 4. Conclusions

Here, the focus has been on developing a surface modification strategy on both porous filter materials (e.g., air filtration units, face masks) and also on solid surfaces against enveloped RNA viruses, such as the SARS-CoV-2 virus. These strategies are based on antimicrobial nanoparticle coatings made by aerosol self-assembly that may be either easily included during the product manufacturing or applied on the products after their fabrication. Three nanomaterials that have exhibited antimicrobial properties, namely, silver, copper oxide and zinc oxide have been explored. These nanoparticle coatings were fabricated by flame aerosol direct deposition, a highly scalable and reproducible nanomanufacturing process that combines particle production and nanocoating assembly in a single step. Nanocoatings were deposited on both flat solid substrates by thermophoresis as well as on filter materials by filtration to exemplify the versatility of this nanomanufacturing process. The morphological and physicochemical properties of these nanoparticle coatings were characterized, and finally their antiviral capacity against SARS-CoV-2 was evaluated by plaque assay.

Among the three nanomaterials studied here, nanosilver exhibited the highest antiviral activity against SARS-CoV-2, reducing the viral load up to 75% after 5 min and 98% after 120 min. These results provide proof-of-principle and encourage further development of this technology to find more potently inhibiting materials. Nanosilver is a well-known antibacterial nanomaterial and is also reported to inhibit several viruses, including the herpes simplex virus 2, hepatitis B virus, Tacaribe virus, vaccinia virus and H1N1 influenza A virus [20,21,22,23,24]. The antimicrobial activity of nanosilver is largely attributed to the released Ag^+^ ions upon its exposure to an aqueous environment, with up to 40% Ag^+^ ion released [25] for the size employed here, and it has further been shown that it targets the bacterial cell envelope by generating reactive oxygen species [10]. SARS-CoV-2 is an enveloped virus, and thus the antiviral activity might be attributed to the same mechanism, even though it has also been shown that Ag^+^ ions inhibit viruses by binding to viral proteins or directly lysing membranes [26,27,28,29]. The antimicrobial activity of copper oxide is also attributed to the generation of reactive oxygen species, but surface-related catalytic activity might also contribute [19]. During the incubation of the nanocoatings with the virus-containing droplet, the virus particles may be inactivated both by their direct interaction with the surface, and by their interaction with the released ions (Ag^+^, Cu^2+^ and Zn^2+^). Upon considering the transport of the virus particles in the liquid solution and the transport of the released ions based on their diffusion coefficients (Stokes–Einstein equation), the virus particle transportation is much slower than that of ions, indicating that any antiviral effect from the ions would occur faster. The outcome of this research provides the necessary guidance to the healthcare sector for utilizing anti-viral coatings on solid surfaces against fomite transmission leading to super-spreading events as well as for mitigating aerosol transmission by utilizing nanosilver-based air filtration units for the current COVID-19 pandemic and any future pandemic caused by similar pathogens. Future studies should focus on further physicochemical parameters of antiviral nanoparticles, such as size and composition, including stimuli-responsive nanomaterials regarding the triggered on-demand disinfection of surfaces.

## Data Availability

The data presented in this study are available on request from the corresponding authors.

## Supplementary Materials

The following are available online at https://www.mdpi.com/article/10.3390/nano11051312/s1. Figure S1: Representative photograph of the plaque quantification assay for the data presented in Figure 4A,B, Table S1: p-values of data in Figure 4a.

## References

[1] World Health Organization Scientific Brief. Transmission of SARS-CoV-2: Implications for Infection Prevention Precautions. https://www.who.int/publications/i/item/modes-of-transmission-of-virus-causing-covid-19-implications-for-ipc-precaution-recommendations.

[2] Van Doremalen N., Bushmaker T., Morris D.H., Holbrook M.G., Gamble A., Williamson B.N., Tamin A., Harcourt J.L., Thornburg N.J., Gerber S.I. (2020). Aerosol and Surface Stability of SARS-CoV-2 as Compared with SARS-CoV-1. N. Engl. J. Med..

[3] Weiss C., Carriere M., Fusco L., Capua I., Regla-Nava J.A., Pasquali M., Scott J.A., Vitale F., Unal M.A., Mattevi C. (2020). Toward Nanotechnology-Enabled Approaches against the COVID-19 Pandemic. ACS Nano.

[4] Verdenelli M.C., Cecchini C., Orpianesi C., Dadea G.M., Cresci A. (2003). Efficacy of antimicrobial filter treatments on microbial colonization of air panel filters. J. Appl. Microbiol..

[5] Sotiriou G.A., Pratsinis S.E. (2011). Engineering nanosilver as an antibacterial, biosensor and bioimaging material. Curr. Opin. Chem. Eng..

[6] Vincent M., Duval R.E., Hartemann P., Engels-Deutsch M. (2018). Contact killing and antimicrobial properties of copper. J. Appl. Microbiol..

[7] Sirelkhatim A., Mahmud S., Seeni A., Kaus N.H.M., Ann L.C., Bakhori S.K.M., Hasan H., Mohamad D. (2015). Review on Zinc Oxide Nanoparticles: Antibacterial Activity and Toxicity Mechanism. Nano. Micro. Lett..

[8] Sotiriou G.A., Pratsinis S.E. (2010). Antibacterial activity of nanosilver ions and particles. Environ. Sci. Technol..

[9] Sotiriou G.A., Meyer A., Knijnenburg J.T.N.N., Panke S., Pratsinis S.E. (2012). Quantifying the Origin of Released Ag^+^ Ions from Nanosilver. Langmuir.

[10] Gunawan C., Faiz M.B., Mann R., Ting S.R.S., Sotiriou G.A., Marquis C.P., Amal R. (2020). Nanosilver Targets the Bacterial Cell Envelope: The Link with Generation of Reactive Oxygen Radicals. ACS Appl. Mater. Interfaces.

[11] Jeremiah S.S., Miyakawa K., Morita T., Yamaoka Y., Ryo A. (2020). Potent antiviral effect of silver nanoparticles on SARS-CoV-2. Biochem. Biophys. Res. Commun..

[12] Balagna C., Perero S., Percivalle E., Nepita E.V., Ferraris M. (2020). Virucidal effect against Coronavirus SARS-CoV-2 of a silver nanocluster/silica composite sputtered coating. Open Ceram..

[13] Mädler L., Stark W.J., Pratsinis S.E. (2002). Rapid synthesis of stable ZnO quantum dots. J. Appl. Phys..

[14] Tricoli A., Graf M., Mayer F., Kuühne S., Hierlemann A., Pratsinis S.E. (2008). Micropatterning Layers by Flame Aerosol Deposition-Annealing. Adv. Mater..

[15] Mädler L., Kammler H.K., Mueller R., Pratsinis S.E. (2002). Controlled synthesis of nanostructured particles by flame spray pyrolysis. J. Aerosol. Sci..

[16] Teoh W.Y., Amal R., Mädler L. (2010). Flame spray pyrolysis: An enabling technology for nanoparticles design and fabrication. Nanoscale.

[17] Strobel R., Pratsinis S.E. (2007). Flame aerosol synthesis of smart nanostructured materials. J. Mater. Chem..

[18] Matrosovich M., Matrosovich T., Garten W., Klenk H.-D. (2006). New low-viscosity overlay medium for viral plaque assays. Virol. J..

[19] Hosseini M., Chin A.W.H., Behzadinasab S., Poon L.L.M., Ducker W.A. (2021). Cupric Oxide Coating That Rapidly Reduces Infection by SARS-CoV-2 via Solids. ACS Appl. Mater. Interfaces.

[20] Hu R.L., Li S.R., Kong F.J., Hou R.J., Guan X.L., Guo F. (2014). Inhibition effect of silver nanoparticles on *herpes simplex* virus 2. Genet. Mol. Res..

[21] Lu L., Sun R.W.-Y., Chen R., Hui C.-K., Ho C.-M., Luk J.M., Lau G.K.K., Che C.-M. (2008). Silver nanoparticles inhibit *hepatitis B* virus replication. Antivir. Ther..

[22] Speshock J.L., Murdock R.C., Braydich-Stolle L.K., Schrand A.M., Hussain S.M. (2010). Interaction of silver nanoparticles with *Tacaribe virus*. J. Nanobiotechnol..

[23] Trefry J.C., Wooley D.P. (2013). Silver Nanoparticles Inhibit *Vaccinia Virus* Infection by Preventing Viral Entry Through a Macropinocytosis-Dependent Mechanism. J. Biomed. Nanotechnol..

[24] Xiang D., Chen Q., Pang L., Zheng C. (2011). Inhibitory effects of silver nanoparticles on H1N1 influenza A virus in vitro. J. Virol. Methods.

[25] Valentini P., Fiammengo R., Sabella S., Gariboldi M., Maiorano G., Cingolani R., Pompa P.P. (2013). Gold-nanoparticle-based colorimetric discrimination of cancer-related point mutations with picomolar sensitivity. ACS Nano.

[26] Borkow G., Gabbay J. (2005). Copper as a Biocidal Tool. Curr. Med. Chem..

[27] Hodek J., Zajícová V., Lovětinská-Šlamborová I., Stibor I., Müllerová J., Weber J. (2016). Protective hybrid coating containing silver, copper and zinc cations effective against human immunodeficiency virus and other enveloped viruses. BMC Microbiol..

[28] Rai M., Yadav A., Gade A. (2009). Silver nanoparticles as a new generation of antimicrobials. Biotechnol. Adv..

[29] Lara H.H., Garza-Treviño E.N., Ixtepan-Turrent L., Singh D.K. (2011). Silver nanoparticles are broad-spectrum bactericidal and virucidal compounds. J. Nanobiotechnol..

