# Supplementary material for: Antiviral Activity of Silver, Copper Oxide and Zinc Oxide Nanoparticle Coatings against SARS-CoV-2
Source: Nanomaterials (Basel). 2021 May 17;11(5):1312. doi: 10.3390/nano11051312 (PMC8155969; doi:10.3390/nano11051312)
Supplement: Supplementary file 1 [file nanomaterials-11-01312-s001.zip › nanomaterials-1142453-supplementary.pdf]

Supplementary Information

Antiviral activity of silver, copper oxide and zinc oxide nanoparticle coatings against SARS-CoV-2

Padryk Merkl[#], Siwen Long[#], Gerald M. McInerney*, and Georgios A. Sotiriou*

Department of Microbiology, Tumor and Cell Biology, Karolinska Institutet, SE-17177 Stockholm, Sweden.
[#]These authors contributed equally
*Corresponding authors E-mails: georgios.sotiriou@ki.se and gerald.mcinerney@ki.se

Table S1. p-values of data in Fig. 4a.

p-values of data vs control in Fig. 4a			
	5 min	30 min	120 min
ZnO	0.4792	0.2061	0.6697
CuO	0.3068	0.008	0.2773
Ag	0.0251	0.0003	0.166

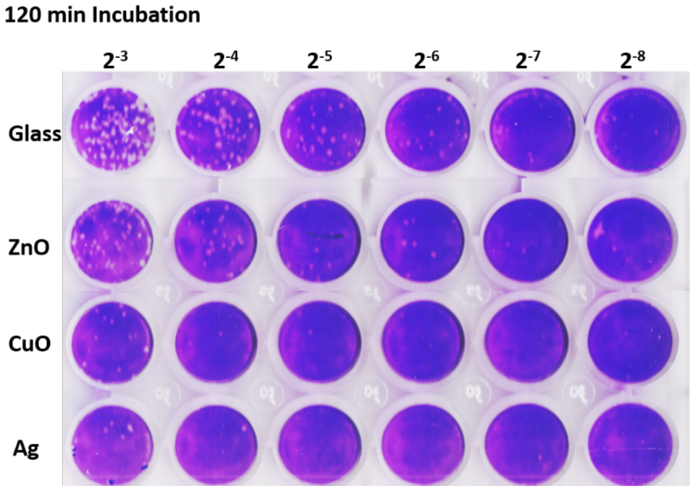

Figure S1. Representative photograph of the plaque quantification assay for the data presented in Fig 4A and 4B.
